# Diagnostic accuracy of the different hormonal tests used for the diagnosis of autonomous cortisol secretion

**DOI:** 10.1038/s41598-021-00011-4

**Published:** 2021-10-15

**Authors:** Marta Araujo-Castro, Ana García Cano, Lucía Jiménez Mendiguchía, Héctor F. Escobar-Morreale, Pablo Valderrábano

**Affiliations:** 1 Neuroendocrinology Unit, Department of Endocrinology & Nutrition, Hospital Universitario Ramón y Cajal, Madrid, Spain; 2 Department of Biochemistry, Hospital Universitario Ramón y Cajal, Madrid, Spain; 3 Department of Endocrinology & Nutrition, Hospital Universitario Ramón y Cajal, Madrid, Spain; 4 Universidad de Alcalá, Madrid, Spain; 5 Instituto Ramón y Cajal de Investigación Sanitaria (IRYCIS), Madrid, Spain; 6 Centro de Investigación Biomédica en Red Diabetes y Enfermedades Metabólicas Asociadas (CIBAERDEM), Madrid, Spain

**Keywords:** Biochemistry, Endocrinology, Urology

## Abstract

To evaluate the diagnostic accuracy of the different tests commonly used in the evaluation of adrenal incidentalomas (AIs) for the identification of autonomous cortisol secretion (ACS) and comorbidities potentially related to ACS. In a retrospective study of patients with AIs ≥ 1 cm, we evaluated the diagnostic reliability and validity of the dexamethasone suppression test (DST), urinary free cortisol (UFC), ACTH, late-night salivary cortisol (LNSC), and dehydroepiandrosterone-sulphate (DHEAS) for the diagnosis of comorbidities potentially related to ACS. Diagnostic indexes were also calculated for UFC, ACTH, LNSC, and DHEAS considering DST as the gold standard test for the diagnosis of ACS, using three different post-DST cortisol thresholds (138 nmol/L, 50 nmol/L and 83 nmol/L). We included 197 patients with AIs in whom the results of the five tests abovementioned were available. At diagnosis, 85.9% of patients with one or more AIs had any comorbidity potentially related to ACS, whereas 9.6% had ACS as defined by post-DST cortisol > 138 nmol/L. The reliability of UFC, ACTH, LNSC, and DHEAS for the diagnosis of ACS was low (kappa index < 0.30). Of them, LNSC reached the highest diagnosis accuracy for ACS identification (AUC = 0.696 [95% CI 0.626–0.759]). The diagnostic performances of these tests for comorbidities potentially related to ACS was poor; of them, the DST was the most accurate (AUC = 0.661 [95% CI 0.546–0.778]) and had the strongest association with these comorbidities (OR 2.6, *P* = 0.045). Patients presenting with increased values of both DST and LNSC had the strongest association with hypertension (OR 7.1, *P* = 0.002) and with cardiovascular events (OR 3.6, *P* = 0.041). In conclusion, LNSC was the test showing the highest diagnosis accuracy for the identification of ACS when a positive DST was used as the gold standard for its diagnosis. The DST test showed the strongest association with comorbidities potentially related to ACS. The definition of ACS based on the combination of elevated DST and LNSC levels improved the identification of patients with increased cardiometabolic risk.

## Introduction

Adrenal incidentalomas (AIs) are defined as adrenal masses detected in imaging tests performed for reasons unrelated to adrenal disease^1–3^. All patients with AIs must be evaluated to exclude malignancy and hormonal excess^4,5^. Even though imaging tests offer a high sensitivity and a reasonable specificity for the diagnosis of malignancy, functional evaluation of AIs is often challenging. Particularly, consensus is lacking regarding the definition and diagnostic criteria of autonomous cortisol secretion (ACS), which may associate an increased cardiometabolic morbidity and mortality and might appear in as many as 20% of patients with AIs^6^. Nonetheless, ACS is usually defined by an incomplete cortisol suppression in response to the overnight 1 mg dexamethasone suppression test (DST), in the absence of clinical data specific of Cushing’s syndrome^4,5,7,8^. Nevertheless, other tests such as 24-h urinary free cortisol (UFC), late-night salivary cortisol (LNSC) and plasma adrenocorticotropic hormone (ACTH) have been proposed for the definition of ACS. However, there are few data comparing these tests and the DST for the diagnosis of ACS; hence, UFC, LNSC and ACTH are usually used as tools to complement the results of the DST in this setting. On the other hand, under usual routine clinical practice conditions, the diagnostic performance of the DST and complementary tests for the identification of comorbidities potentially related to ACS seems to be poor.

We hypothesized that the identification of cardiometabolic morbidities potentially related to ACS in patients with AIs could improve with the use of a panel of tests usually used to characterize adrenal function, either individually or in combination. Moreover, we evaluated the reliability and validity for the diagnosis of ACS—considering an increased DST result as the gold standard for ACS definition following current European clinical guidelines^2^—of four tests routinely used for the evaluation of adrenal function, including plasma ACTH, age and sex adjusted serum dehydroepiandrosterone sulphate (DHEA-S) levels, UFC and LNSC.

## Methods

### Patients

**Figure 1:**
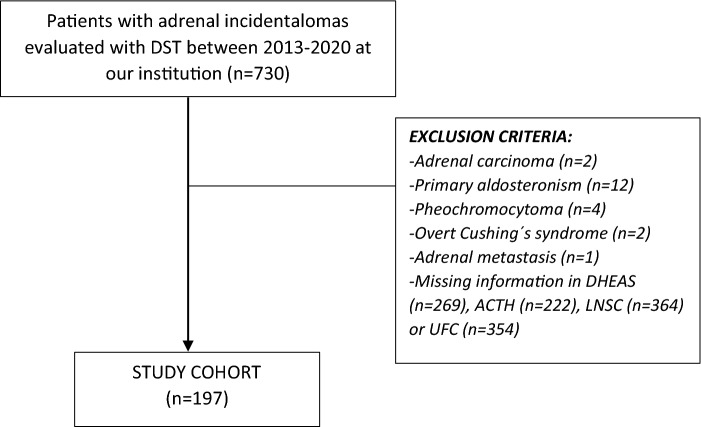
Study cohort. *DST* dexamethasone suppression test, *LNSC* late-night salivary cortisol, *UFC* urinary free cortisol.

We retrospectively queried the electronic registry of the hormone laboratory of Hospital Universitario Ramón y Cajal to identify all patients in whom a DST had been performed between 2013 and 2020. We reviewed their medical records and selected those patients aged 18 to 90 years-old who presented with incidentally discovered unilateral and/or bilateral AIs of at least 10 mm in the largest diameter. We excluded patients with: (i) known diagnosis of hereditary syndromes associated with adrenal tumours; (ii) chronic treatment with glucocorticoids or drugs that might affect dexamethasone metabolism; (iii) treatment with oral hormonal contraceptives during the 6 weeks preceding the test; (iv) AIs identified during the extension study of an extra-adrenal cancer; (v) patients with overt syndromes of adrenal hormone excess, (vi) adrenocortical carcinoma; (vii) adrenal metastasis from extra-adrenal tumours; and (viii) missing information in the results of one or more of the five tests evaluated here) (Fig. 1). We analysed patients’ data obtained during their initial evaluation and at their last available follow-up visit.

### Clinical evaluation

Demographics information such as age and sex; presence of comorbidities potentially related to ACS (hypertension, type 2 diabetes, obesity, dyslipidaemia, cerebrovascular and cardiovascular disease); body mass index (BMI); and systolic and diastolic blood pressure were extracted from medical records. Obesity was defined by a BMI equal or greater to 30 kg/m^2^. Hypertension was defined as systolic blood pressure equal to or greater than 140 mmHg and/or diastolic blood pressure equal to or greater than 90 mmHg, or treatment with blood pressure lowering medications. Diagnosis of type 2 diabetes and dyslipidaemia was based on current standards^9,10^. Cardiovascular disease was defined as ischemic heart disease or heart failure, and cerebrovascular disease as transient ischemic attack or acute stroke.

Management decision regarding AIs—either observation or surgery—after the last follow-up visit was also registered.

### Biochemical and hormonal evaluation

Routine biochemical profile after an 8 h overnight fasting was performed at diagnosis and at the last follow-up visit available. Biochemical profiles included fasting plasma glucose, total cholesterol, LDL-cholesterol, HDL-cholesterol, triglycerides and HbA1c (the latter was available only in 55 cases). Hormonal studies at the initial evaluation included urinary catecholamines and/or urinary metanephrines, DST, UFC, ACTH, DHEA-S and LNSC.

DST, UFC, ACTH, age- and sex-adjusted DHEA-S, and LNSC were analysed as continuous and categorical variables. When considering the DST test as the gold standard for the calculation of reliability and validity for ACS diagnosis of the others tests of adrenal function, we evaluated not only the post-DST cortisol 138 nmol/L (5.0 µg/dL)^2^, but also the 50 nmol/L (1.8 µg/dL) and 83 nmol/L (3.0 µg/dL) cut-off values. For the evaluation of the diagnosis accuracy of the DST for the identification of comorbidities potentially related to ACS, the > 50 nmol/L threshold was employed, based on the results of the ROC curves and on previous studies that found that this cut-off was the most sensitive for this purpose^11–15^. UFC levels above the upper limit of the reference range in our laboratory were considered elevated. Besides, patients with UFC levels within the reference range were classified into two groups—normal-low or normal-high UFC levels—using 1930 nmol/24 h (70 µg/24 h) as threshold, because this was the value that associated the highest specificity for the diagnosis of ACS according to the results of the ROC curve. Patients with UFC levels two-fold above the reference range were diagnosed with overt Cushing’s syndrome and excluded from the study (Fig. 1). ACTH levels below 2 pmol/L (10 pg/mL) were considered low. LNSC levels above the upper limit of the reference range in our laboratory were considered elevated. DHEA-S levels were considered to be elevated or decreased according to age- and sex-specific reference ranges in our laboratory.

### Laboratory assays

As we have previously reported^16^, urine and serum cortisol were measured by immunochemiluminescence assays in an Architect i2000 systems Abbott Diagnostics platform, with an intra-assay coefficient of variation (CV) < 10%; the normal range was 102–535 nmol/L (3.7–19.4 µg/dL) for serum cortisol and < 3862 nmol/24 h (140 µg/24 h) for 24-h urine cortisol. LNSC was measured by electroimmunochemiluminescence in a Cobas 6000 Roche autoanalyser, with an intra-assay CV < 10% and a reference range lower than 157 nmol/L (< 5.7 µg/dL). The determination of ACTH was performed by immunochemiluminescence assays (we used Immulite 2000 Siemens before 2019 and Liaison XL Diasorin thereafter), with an intra-assay CVs < 10%. Normal values for ACTH were between 2.0–10.1 pmol/L (9–46 pg/mL) for the Immulite assay and 1.0–10.7 pmol/L (5–49 pg/mL) for the Liaison XL assay. DHEA-S was measured by immunochemiluminescence assay in Immulite 2000 Siemens system; with intra-assay CV < 15%. Reference ranges for DHEA-S were age- and sex-specific (Table 1).

**Table 1:** References ranges for DHEAS levels (ng/mL).

Age	Female ( ng/mL)	Age	Males ( ng/mL)
18–24	150–3402	20–29	2800–6400
25–34	150–2982	30–39	1200–5200
35–49	150–2582	40–49	950–5300
50–59	260–2000	50–59	700–3100
60–69	130–1300	60–69	420–2900
70–79	280–1750	70–89	280–1750

### Imaging studies

At diagnosis, abdominal computed tomography or magnetic resonance imaging were obtained in all AIs patients. Tumour size (largest diameter), uni- or bilaterality, presence of necrosis, calcification and atypical characteristics, lipid content and radiodensity measured in Hounsfield units (HU) were registered. In bilateral AIs, the recorded tumour size was that of the largest AI. The adrenal tumour was classified as having rich lipid content when attenuation was low (< 10 HU) in a CT performed without contrast administration or when the washout in a CT with contrast was rapid (> 60% absolute washout or > 40% relative washout)^4^. Computed tomography was repeated in 99 patients and magnetic resonance imaging was repeated in 80 patients during follow-up.

### Statistical analysis

We checked continuous variables for normality using the Shapiro–Wilk test, and for homogeneity of the variances using Levene’s test. Categorical variables were expressed as counts and percentages, whereas continuous variables were expressed as mean ± standard deviation or median and interquartile range (IQR) as appropriate. Odds ratios (with 95% confidence intervals) and mean differences were calculated as association measures using logistic regression models or lineal regression β coefficients. For variables following the normal distribution, we used Student’s t test to compare differences between two groups. The chi-square test was used for the comparison of categorical variables between independent groups. Cox regression analysis was used to estimate hazard ratios during follow-up. Reliability was evaluated with the kappa index and the specific positive and negative agreement indexes. Nonparametric receiver-operator curve (ROC) analysis was used to determine the diagnostic accuracy for the diagnosis of ACS, and of comorbidities potentially related to ACS, of the different hormonal tests, either individually or in combination. In all cases, a two-tailed *P* value < 0.05 was considered as statistically significant. All statistical analyses were performed using STATA 15 (StataCorp. 2017. Stata Statistical Software: Release 15. College Station, TX: StataCorp LLC).

### Ethical approval

All procedures performed in the participants of the study were in accordance with the ethical standards of the institutional research committee and with the 1964 Helsinki declaration and its later amendments or comparable ethical standards. The study was approved by the Ethics Committee of Hospital Universitario Ramón y Cajal on February 14, 2019.

### Informed consent

The Ethical committee of Hospital Universitario Ramón y Cajal approved the waiver for informed consent given the retrospective nature of the study.

## Results

### Cardiometabolic profile at diagnosis and during follow-up

Following inclusion and exclusion criteria, 197 patients—of a total of 709 patients with AIs consecutively evaluated between 2013 and 2020 at our centre—were included in the analysis. No statistically significant differences were detected between the patients with AIs included or excluded in the study with the exception of higher cortisol post-DST, lower ACTH levels and a larger tumour size in the former (Supplementary Material Table S1). Baseline characteristics of the cohort included in the present study are summarized in Table 2. At diagnosis, 19 patients (9.6%) had ACS (as defined by a post-DST cortisol > 138 nmol/L) and 169 patients (85.9%) presented with one or more comorbidities potentially related to ACS. The prevalence of obesity was of 31%, yet no statistically significant differences in the post-DST cortisol levels were found between patients with and without obesity (59 ± 49 nmol/L vs 71 ± 82 nmol/L, respectively, P = 0.316). Four patients presenting with non-functioning AIs > 4 cm underwent adrenalectomy, and active surveillance was carried out in the remainder. After a median follow-up of 30.6 (IQR = 2.0–114.7) months, 6 out of 120 patients with non-functioning AIs developed ACS and 23 patients developed one or more new comorbidities: 20 (23.0%) developed dyslipidaemia; 6 (8.8%) developed hypertension; 9 (11.5%) became obese; 6 (4.5%) were diagnosed with type 2 diabetes; and 5 (3.2%) suffered a cardiovascular event. No cerebrovascular events were registered during follow-up.

**Table 2:** Baseline characteristics of the cohort (n = 197).

Parameter	Value
**Clinical data**	
Age, years	64.5 ± 10.0
Female sex	57.4% (n = 113)
Comorbidities potentially related to ACS	85.9% (n = 152)
Diabetes	22.3% (n = 44)
Hypertension	57.9% (n = 114)
Dyslipidaemia	49.0% (n = 96)
Obesity	31.0% (n = 61)
Cerebrovascular disease	1.0% (n = 2)
Cardiovascular disease	10.7% (n = 21)
Body mass index (kg/m^2^) (n = 133)	30.3 ± 6.3
Systolic blood pressure (mmHg) (n = 159)	137.7 ± 16.9
Diastolic blood pressure (mmHg) (n = 159)	79.7 ± 9.6
**Analytical data**	
Fasting plasma glucose, nmol/L (mg/dL) (n = 197)	5.87 ± 1.6 (105.7 ± 28.9)
HbA1c (%) (n = 55)	6.2 ± 0.9
LDL-c, nmol/L (mg/dL) (n = 143)	30.03 ± 8.3 (115.5 ± 31.8)
HDL-c, nmol/L (mg/dL) (n = 143)	13.96 ± 4.6 (53.7 ± 17.7)
Triglycerides, nmol/L (mg/dL) (n = 193)	1.17 ± 0.6 (110.2 ± 51.1)
DST, nmol/L(µg/dL) (n = 197)	66.2 ± 74.5 (2.4 ± 2.7)
Urinary free cortisol, nmol/24 h (µg/24 h) (n = 197)	1092.41 ± 791.1 (39.6 ± 28.7)
ACTH, pmol/L (pg/mL) (n = 197)	3.59 ± 2.6 (16.3 ± 11.6)
DHEAS (ng/mL) (n = 197)	596.2 [IQR = 150–2840]
Late-night salivary cortisol, nmol/L(µg/dL) (n = 197)	110.3 ± 118.6 (4.0 ± 4.3)
**Radiological data**	
Tumor size (mm) (n = 197)	22.2 ± 10.5
Bilaterality (n = 197)	30.0% (n = 59)
Tumor rich in lipidic content (n = 155)	85.2% (n = 132)

*ACTH* adrenocorticotropic hormone, *ACS* autonomous cortisol secretion, *DST* dexamethasone suppresion test, *DHEAS* dehydroepiandrosterone sulphate, *LDL-c* low-density lipoprotein cholesterol, *HDL-c* high-density lipoprotein cholesterol, *HbA1c* hemoglobin A1c.

### Reliability and accuracy of LNSC, UFC, ACTH and DHEAS for the diagnosis of ACS

**Figure 2:**
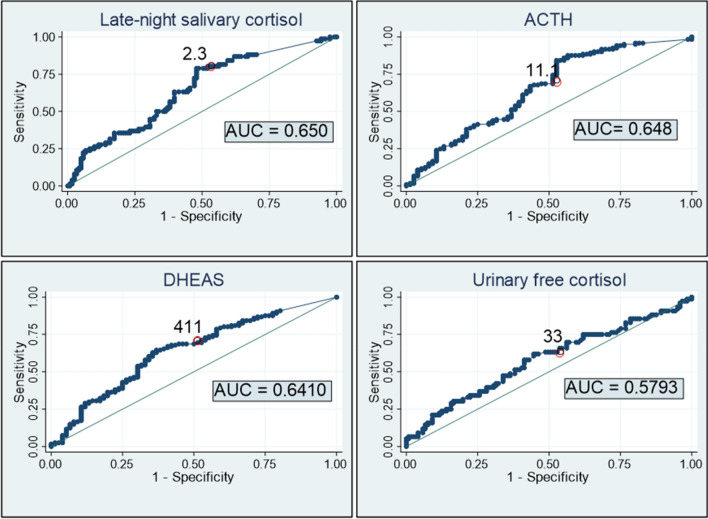
Diagnosis accuracy of LNSC, ACTH, DHEAS and UFC for the diagnosis of ACS (considering the 1.8 µg/dL threshold for the diagnosis of Autonomous cortisol secretion). Optimal cutoff point based on ROC curve 2.26 µg/dL (Sensitivity: Se = 78.9% (95% CI 68.5 to 86.6) and Specificity: Sp = 52.1% (95% CI 43.2 to 60.8). *ACTH* AUC 0.648, 95% CI 0.577–0.715. Optimal cutoff point based on ROC curve: 11.08 pg/mL (Se = 66.9% (95% CI 58.2 to 74.7) and Sp = 56.6% (95% CI 45.4 to 67.1). *DHEA-S* AUC 0.640, 95% CI 0.570–0.708. Optimal cutoff point based on ROC curve: 411 µg/dL (Se = 64.5% (95% CI 55.6 to 72.4) and Sp = 63.2% (95% CI 51.9 to 73.1). *24 h-urinary free cortisol (UFC)* AUC 0.579, 95%CI 0.507–0.649. Optimal cutoff point based on ROC curve 32.6 µg/24 h (Se = 61.8% (95% CI 50.6 to 71.9 and Sp = 55.4% (95% CI 46.5 to 63.9).

**Figure 3:**
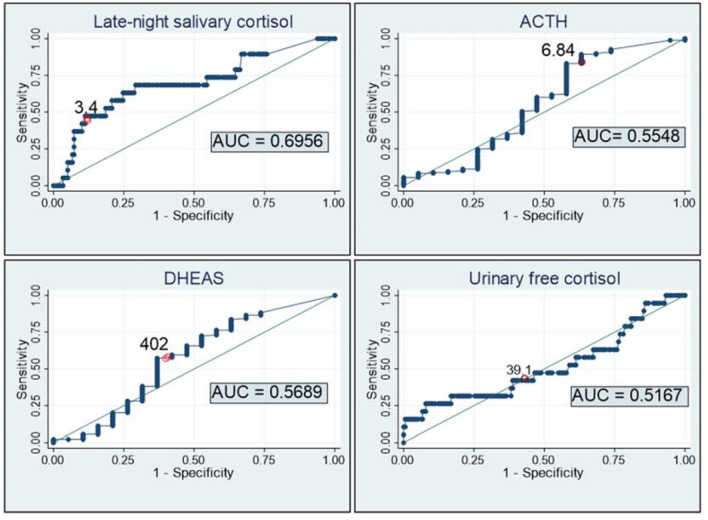
Diagnosis accuracy of LNSC, ACTH, DHEAS and UFC for the diagnosis of ACS (considering the 5 µg/dL threshold for the diagnosis of Autonomous cortisol secretion). *Late-night salivary cortisol* AUC = 0.696 (95% CI 0.626 to 0.759). Optimal cut-off point based on ROC curve: 3.4 µg/dL (Se = 68.4% (95% CI 46.0 to 84.6) and Sp = 70.8% (95% CI 63.7 to 77.0). *ACTH* AUC = 0.555 (95% CI 0.483 to 0.625). Optimal cut-off point based on ROC curve: 6.84 pg/mL (Se = 83.1% (95% CI 77.0 to 87.9) and Sp = 42.1% (95% CI 23.1 to 63.7). *DHEAS* AUC = 0.569 (95% CI 0.497 to 0.639). Optimal cut-off point based on ROC curve: 402 µg/dL (Se = 57.3% (95% CI 50.0 to 64.3) and Sp = 63.2% (95% CI 41.0 to 80.9). *UFC* AUC 0.517 (95% CI 0.445 to 0.588). Optimal cut-off point based on ROC curve: 39.1 µg/24 h (Se = 42.1% (95% CI 23.1 to 63.7) and Sp = 61.2% (95% CI 53.9 to 68.1).

The degree of agreement (reliability) of LNSC, UFC, ACTH and DHEA-S for the diagnosis of ACS was low, independently of the DST threshold used for the definition of ACS, with kappa indexes below 0.3 for all tests. However, the specific negative agreement was high, around 80–90%. Regarding their validity, the highest specificity was reached when ACS definition was based on the 138 nmol/L (5.0 µg/dL) threshold. Nevertheless, all tests had poor sensitivity for the diagnosis of ACS independently of the DST threshold employed for the diagnosis of ACS (Table 3). ROC curves confirmed these findings, supporting that these tests should not be used in isolation for the diagnosis of ACS. The greatest diagnostic accuracy, although modest, was that of LNSC (Figs. 2, 3). Moreover, when the four tests were combined, the diagnostic accuracy for the diagnosis of ACS increased, reaching an AUC of 0.73 [0.65–0.80].

**Table 3:** Reliability and validity of LNSC, UFC, ACTH and DHEAS for the diagnosis of ACS (considering three different thresholds in the DST for the ACS definition).

	Kappa index	Specific Po+ (%)	Specific Po− (%)	Sensitivity (%)	Specificity (%)	PPV (%)	NPV (%)
**Considering the gold standard of ACS a DST > 138 nmol/L (5.0 µg/dL)**							
UFC > 1931 nmol/24 h	0.157	24.4	91.2	26.3	90.4	22.7	92.0
ACTH < 2 pmol/L	0.039	18.4	76.9	42.1	66.3	11.8	91.5
LNSC > 157 nmol/L	0.283	36.7	91.0	47.4	88.2	30.0	94.0
Low sex- and age- adjusted DHEA-S	0.082	20.9	83.8	36.8	77.0	14.6	91.9
**Considering the gold standard of ACS a DST > 83.0 nmol/L (3.0 µg/dL)**							
UFC > 1931 nmol/24 h	0.239	34.9	87.6	26.8	92.9	50.0	82.9
ACTH < 2 pmol/L	0.145	36.7	75.8	48.8	69.2	29.4	83.7
LNSC > 157 nmol/L	0.231	36.6	86.1	31.7	89.1	43.3	83.2
Low sex- and age-adjusted DHEA-S	0.116	31.5	80.0	34.1	78.2	29.2	81.9
**Considering the gold standard of ACS a DST > 50.0 nmol/L (1.8 µg/dL)**							
UFC > 1931 nmol/24 h	0.086	24.5	75.0	15.8	91.7	54.5	63.4
ACTH < 2 pmol/L	0.235	51.4	72.0	48.7	74.4	54.4	69.8
LNSC > 157 nmol/L	0.179	35.8	76.4	25.0	90.9	63.3	65.9
Low sex- and age-adjusted DHEA-S	0.103	37.1	71.1	30.3	79.3	47.9	64.4

*ACTH* adrenocorticotropic hormone, *DST* dexamethasone suppression test, *DHEAS* dehydroepiandrosterone sulphate, *LNSN* late-night salivary cortisol, *PPV* positive predictive value, *NPV* negative predictive value, *Specific Po* + specific positive agreement index, *Specific Po − *specific negative agreement index, *UFC* urinary-free cortisol.

### Association of the individual ACS diagnostic tests’ results with comorbidities potentially related to ACS

**Figure 4:**
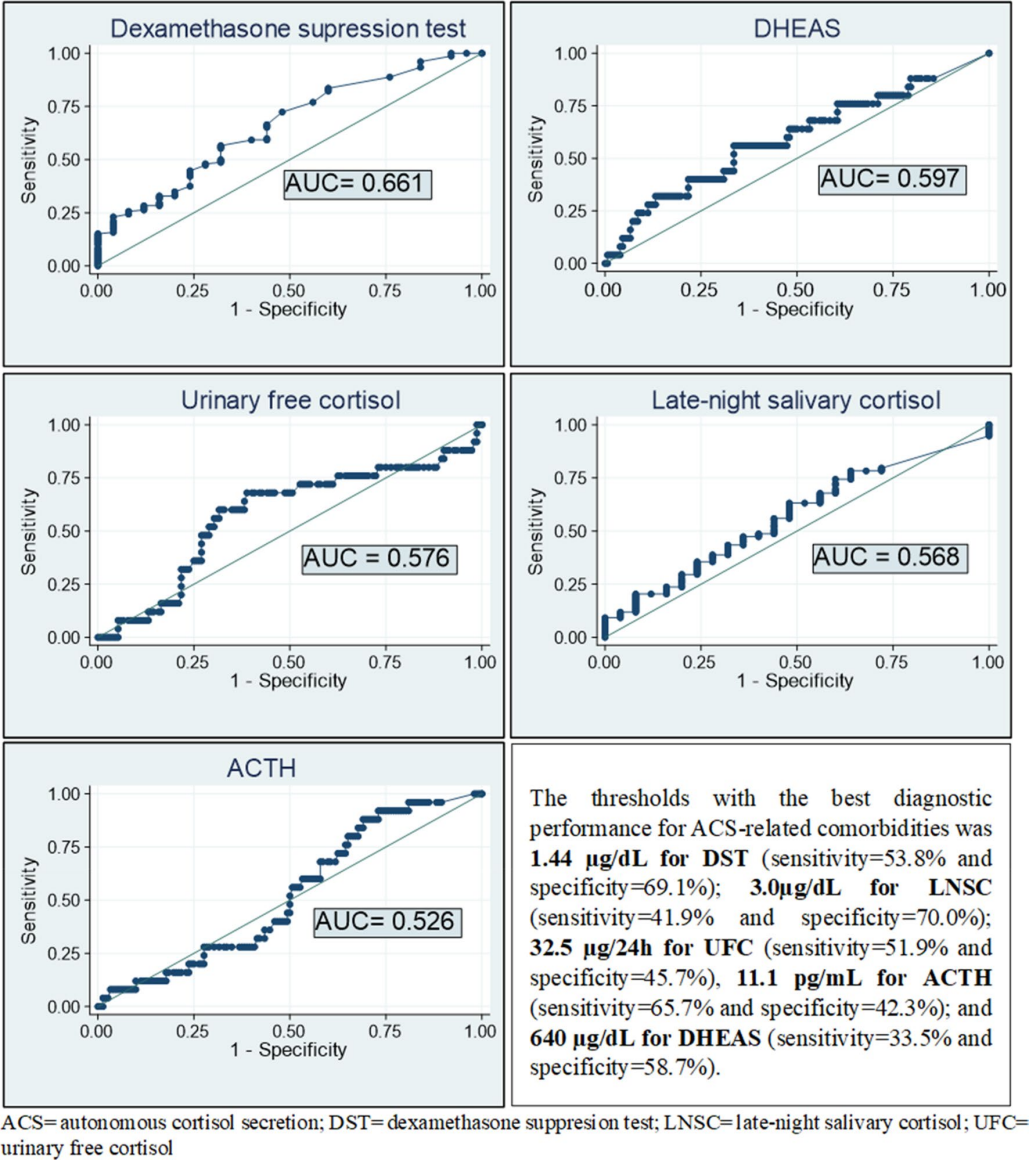
ROC curve of the different tests for the diagnosis of any comorbidities potentially related to ACS.

Seventy-six (38.6%) patients showed a DST serum cortisol level > 50 nmol/L (1.8 µg/dL) at diagnosis. These patients had a risk of comorbidities potentially related to ACS two-fold higher than those with DST ≤ 50 nmol/L. The prevalence of dyslipidaemia and hypertension in patients with DST > 50 nmol/L was 1.8 and 2.5 times higher than in patients with DST ≤ 50 nmol/L, respectively (Table 4). However, the diagnostic performance of the DST to predict the presence of one or more comorbidities potentially related to ACS either individually or collectively, was poor, because all areas under the ROC curve analyses were below 0.67) (Fig. 4).

**Table 4:** Baseline features and association of ACS-diagnostic tests with the diagnosis of comorbidities potentially related to ACS.

	DST (nmol/L) > 50 (n = 76) vs ≤ 50 (n = 121)	UFC (nmol/24 h) ≥ 1930 (n = 22) vs < 1930 (n = 175)	ACTH (pmol/L) < 2 (n = 68) vs ≥ 2 (n = 129)	DHEAS (µg/dL) low (n = 48) vs normal (n = 149)	LNSC (nmol/L) > 157 (n = 30) vs ≤ 157 (n = 167)
Age, years	65.7 ± 10.0 vs 63.7 ± 10.1, P = 0.173	64.6 ± 10.0 vs 64.5 ± 10.1, P = 0.943	63.3 ± 9.2 vs 65.1 ± 10.5, P = 0.252	64.3 ± 10.1 vs 64.5 ± 10.1, P = 0.885	66.9 ± 10.8 vs 64.1 ± 9.9, P = 0.154
Male sex	OR 0.8 [0.5–1.4], P = 0.476	OR 1.4 [0.6–3.4], P = 0.461	OR 0.6 [0.3–1.1], P = 0.068	OR 2.3 [1.2–4.5], P = 0.012	OR 1.0 [0.5–2.3], P = 0.934
Comorbidities potentially related to ACS (composite)	OR 2.6[1.0–6.8], P = 0.045	OR 1.7 [0.4–8.0], P = 0.446	OR 1.7 [0.7–4.6], P = 0.248	= 1.0 [0.4–2.7], P = 0.971	OR 2.5 [0.6–11.2], P = 0.188
Hypertension	OR 2.5 [1.4–4.6], P = 0.003	OR 2.7 [1.0–7.7], P = 0.043	OR 1.0 [0.5–1.8], P = 0.915	OR 0.8 [0.4–1.6] P = 0.551	OR 2.7 [1.1–6.7], P = 0.020
Dyslipidaemia	OR 1.8 [1.0–3.2], P = 0.054	OR 1.0 [0.4–2.5], P = 0.940	OR 1.0 [0.6–1.9], P = 0.877	OR 1.0 [0.5–2.0], P = 0.903	OR 1.2 [0.6–2.6], P = 0.626
Obesity	OR 1.0 [0.6–1.9], P = 0.883	OR 2.0 [0.8–5.0], P = 0.130	OR 1.0 [0.5–1.9], P = 0.986	OR 1.0 [0.5–2.1], P = 0.961	OR 0.6 [0.3–1.6], P = 0.316
Diabetes	OR 1.6 [0.8–3.2], P = 0.161	OR 1.7 [0.7–4.6], P = 0.275	OR 1.1[0.6–2.2], P = 0.771	OR 1.0[0.5–2.3], P = 0.912	OR 1.1[0.4–2.7], P = 0.887
Cardiovascular disease	OR 2.3[0.9–5.8], P = 0.069	OR 1.4[0.4–5.1], P = 0.642	OR 0.9[0.4–2.5], P = 0.904	OR 1.6[0.6–4.4], P = 0.326	OR 1.9[0.6–5.6], P = 0.273
Cerebrovascular disease	OR 1.6[0.1–26.0], P = 0.742	NC	OR 1.9[0.1–31.0], P = 0.652	OR 3.1 [0.2–51.3], P = 0.432	NC
Body mass index (kg/m^2^) (n = 133)	30.4 ± 6.6 vs 30.2 ± 6.2, P = 0.866	31.6 ± 5.8 vs 30.2 ± 6.4, P = 0.444	30.5 ± 7.1 vs 30.2 ± 6.0, P = 0.824	30.2 ± 5.4 vs 30.4 ± 6.7, P = 0.932	31.4 ± 7.1 vs 30.2 ± 6.2, P = 0.447
Systolic blood pressure (n = 159)	136.9 ± 16.3 vs 138.2 ± 17.3, P = 0.642	138.8 ± 16.3 vs 137.5 ± 17.0, P = 0.779	135.9 ± 16.1 vs 136.8 ± 17.2, P = 0.352	137.8 ± 18.2 vs 137.6 ± 16.5, P = 0.971	137.2 ± 19.1 vs 137.8 ± 16.5, P = 0.889
Diastolic blood pressure (n = 159)	78.4 ± 9.6 vs 80.7 ± 9.6, P = 0.136	76.9 ± 9.4 vs 80.1 ± 9.6, P = 0.198	77.9 ± 7.6 vs 80.6 ± 10.4, P = 0.095	76.4 ± 9.9 vs 80.9 ± 9.3, P = 0.009	78.6 ± 7.3 vs 79.9 ± 10.0, P = 0.541
Fasting plasma glucose (nmol/L) (n = 197)	6.7 ± 2.3 vs 6.1 ± 1.2 P = 0.015	6.7 ± 2.3 vs 6.3 ± 1.7, P = 0.376	6.3 ± 1.7 vs 6.4 ± 1.8, P = 0.837	6.8 ± 2.5 vs 6.2 ± 1.4, P = 0.053	6.1 ± 1.2 vs 6.4 ± 1.8, P = 0.504
HbA1c (%) (n = 55)	6.4 ± 1.0 vs 6.1 ± 0.8 P = 0.225	6.3 ± 0.8 vs 6.2 ± 0.9, P = 0.799	6.0 ± 0.8 vs 6.3 ± 1.0, P = 0.346	6.2 ± 1.0 vs 6.2 ± 0.9, P = 0.826	6.2 ± 0.7 vs 6.2 ± 1.0, P = 0.820
LDL-c (mmol/L) (n = 143)	29.9 ± 7.8 vs 30.1 ± 8.6 P = 0.860	30.3 ± 9.6 vs 30.0 ± 8.1, P = 0.859	31.4 ± 7.0 vs 29.4 ± 8.8, P = 0.164	28.4 ± 6.7 vs 30.6 ± 8.7, P = 0.148	30.5 ± 9.5 vs 29.9 ± 8.1, P = 0.755
HDL-c (mmol/L) (n = 143)	13.1 ± 4.2 vs 14.5 ± 4.8, P = 0.079	13.6 ± 5.5 vs 14.0 ± 4.5, P = 0.747	13.8 ± 4.0 vs 14.0 ± 4.9, P = 0.835	12.6 ± 4.6 vs 14.4 ± 4.5, P = 0.035	11.7 ± 4.2 vs 14.4 ± 4.6, P = 0.012
Triglycerides (mmol/L) (n = 193)	1.2 ± 0.6 vs 1.1 ± 0.5, P = 0.256	1.0 ± 0.3 vs 1.1 ± 0.5, P = 0.278	1.0 ± 0.1 vs 1.2 ± 0.5, P = 0.033	1.1 ± 0.5 vs 1.1 ± 0.5, P = 0.693	1.1 ± 0.4 vs 1.1 ± 0.5, P = 0.974
DST (nmol/L) (n = 197)	121.0 ± 95.2 vs 33.0 ± 9.13, P < 0.0001	107.4 ± 117.8 vs 61.9 ± 64.3, P = 0.006	75.2 ± 59.0 vs 62.7 ± 79.6, P = 0.253	86.0 ± 105.8 vs 60.9 ± 58.1, P = 0.039	117.5 ± 21.2 vs 57.9 ± 58.6, P < 0.0001
ACTH (pmol/L) (n = 197)	3.0 ± 2.42 vs 4.0 ± 2.42, P = 0.011	4.6 ± 3.1 vs 3.5 ± 2.5, P = 0.056	1.5 ± 0.4 vs 4.7 ± 2.5, P < 0.0001	3.1 ± 1.8 vs 3.8 ± 2.8, P = 0.110	3.2 ± 2.1 vs 3.6 ± 2.6, P = 0.403
UFC (nmol/L) (n = 197)	1256.6 ± 974.0 vs 988.7 ± 635.1, P = 0.020	2811.6 ± 883.5 vs 875.9 ± 883.5, P < 0.0001	983.9 ± 647.3 vs 1149.1 ± 855.2, P = 0.165	1155.7 ± 912.8 vs 1071.5 ± 751.0, P = 0.523	1329.4 ± 1043.9 vs 1049.4 ± 733.5, P = 0.075
DHEAS (µmol/L) (n = 197)	1294.4 ± 1216.8 vs 1821.5 ± 1463.0, P = 0.009	1932.9 ± 1601.0 vs 1578.6 ± 1365.8, P = 0.262	1276.3 ± 1026.8 vs 1798.3 ± 1526.0, P = 0.0121	627.2 ± 329.2 vs 1937.4 ± 1435.3, P < 0.0001	1533.1 ± 1235.4 vs 1633.4 ± 1423.4, P = 0.718
LNSC (nmol/L) (n = 197)	5.0 ± 4.8 vs 3.3 ± 3.9, p = 0.009	147.9 ± 141.3 vs 104.7 ± 115.5, P = 0.109	118.0 ± 118.6 vs 105.1 ± 119.3, P = 0.471	113.4 ± 146.2 vs 108.3 ± 109.3, P = 0.797	330.0 ± 176.2 vs 70.0 ± 30.5, P < 0.0001
Tumor size (mm) (n = 197)	137.3 ± 131.7 vs 92.1 ± 107.1, p < 0.0001	24.2 ± 14.5 vs 22.0 ± 10.0, P = 0.478	25.0 ± 10.8 vs 20.6 ± 10.1, P = 0.021	24.8 ± 10.6 vs 21.3 ± 10.4, P = 0.094	25.4 ± 11.5 vs 21.7 ± 10.3, P = 0.170
Bilaterality (n = 197)	OR 4.3[2.2–8.1], P < 0.001	OR 2.6[1.1–6.5], P = 0.036	OR 2.5 [1.3–4.6], P = 0.005	OR 0.8 [0.4–1.7], P = 0.616	OR 2.0[0.9–4.5], P = 0.091
Tumor rich in lipidic content (n = 155)	OR 1.6 [0.6–4.2], P = 0.303	OR 0.4 [0.1–1.1], P = 0.101	OR 1.7[0.6–4.7], P = 0.267	OR 0.7[0.3–1.7], P = 0.423	OR 0.4[0.1–1.0] P = 0.060

Differences in quantitative variables are expressed in mean differences (d) between ACS and NFAI group, and for qualitative variables differences are expressed in odds ratios (OR) and 95% confident interval (in brackets).

*ACS* autonomous cortisol secretion, *DST* dexamethasone suppression test, *DHEAS* dehydroepiandrosterone sulphate, *NFAI* non-functioning adrenal incidentalomas, *LNSC* late-night salivary cortisol, *UFC* urinary-free cortisol.

UFC was above the > 3862 nmol/24 h in 2 (1.0%) patients whereas another 22 (11.2%) subjects showed normal-high (1931–3862 nmol/24 h) UFC concentrations. The prevalence of hypertension was three times higher in patients with normal-high UFC than in patients with normal-low UFC (< 1931 nmol/24 h) (Table 4). LNSC was above the reference range in 30 (15.2%) patients, who had higher prevalences of hypertension and lower HDL-c levels when compared with patients showing LNSC levels within the reference range (Table 4). Basal ACTH levels were < 2 pmol/L in 68 (34.5%) patients and DHEAS levels were below the age and sex-adjusted reference ranges in 48 (24.4%) patients. No differences were found in the prevalence of ACS-related comorbidities according to ACTH or DHEAS levels. The AUCs for the diagnosis of ACS-related comorbidities were poor for UFC, LNSC, ACTH and DHEAS levels; and do not even reaching that of the DST ROC curve (Fig. 4). Even, when the five tests (including the DST) were used in combination for the prediction of comorbidities potentially related to ACS, the AUC was modest with an AUC of 0.70 [0.58–0.82].

When we evaluated the combined use of the tests for the diagnosis of comorbidities potentially related to ACS, the best association was that of the combination of a DST > 50 nmol/L and a LNSC > 149 nmol/L, which was present in 19 patients in our cohort. These patients had increased risks of hypertension (OR 7.1, 95% CI 1.6–31.6) and cardiovascular events (OR 3.6, 95% CI 1.2–11.3) (Table 5).

**Table 5:** Association of the different combined tests with cardiometabolic comorbidities at presentation.

	DST > 50 nmol/L + LNSC > 149 nmol/L (19 vs. 178)	DST > 50 nmol/L + ACTH < 2 pmol/L (37 vs. 160)	DST > 50 nmol/L + UFC > 1931 nmol/L (12 vs. 185)	DST > 50 nmol/L + low DHEAS (23 vs. 174)	LNSC > 149 nmol/L + ACTH < 2 pmol/L (12 vs. 185)	LNSC > 149 nmol/L + UFC > 1931 nmol/L (6 vs. 191)	LNSC > 149 nmol/L + low DHEAS (8 vs. 189)
Comorbidities potentially related to ACS (any)	OR 1.0	OR 6.9, 95% CI 0.9–53.0, P = 0.013	OR 1.0	OR 3.8, 95% CI 0.5–30.0, P = 0.121	OR 1.0	OR 1.0	OR 1.0
Hypertension	OR 7.1, 95% CI 1.6–31.6, P = 0.0015	OR 1.9, 95% CI 0.9–4.2, P = 0.085	OR 8.8, 95% CI 1.1–69.2, P = 0.007	OR 1.8, 95% CI 0.7–4.5, P = 0.219	OR 2.3, 95% CI 0.6–8.7, P = 0.202	OR 3.8, 95% CI 0.4–32.8, P = 0.175	OR 5.4., 95% CI 0.6–44.5, P = 0.062
Type 2 diabetes	OR 0.6, 95% CI 0.2–2.5, P = 0.455	OR 1.6, 95% CI 0.7–3.6, P = 0.243	OR 1.2, 95% CI 0.3–4.5	OR 1.6, 95% CI 0.6–4.2, P = 0.337	OR 1.2, 95% CI 0.3–4.5, P = 0.821	OR 0.7, 95% CI 0.1–6.1, P = 0.726	OR 1.0
Dyslipidaemia	OR 1.5, 95% CI 0.6–3.8, P = 0.427	OR 1.3, 95% CI 0.6–2.6, P = 0.516	OR 1.2, 95% CI 0.5–4.8	OR 2.6, 95% CI 1.0–6.7, P = 0.036	OR 1.5, 95% CI 0.5–4.8, P = 0.515	OR 1.0, 95% CI 0.2–5.2, P = 0.970	OR 1.8, 95% CI 0.4–7.6, P = 0.442
Obesity	OR 0.4, 95% CI 0.1–1.4, P = 0.111	OR 1.3, 95% CI 0.6–2.7, P = 0.546	OR 1.1, 95% CI 0.3–3.9, P = 0.856	OR 0.8, 95% CI 0.3–2.0, P = 0.585	OR 0.4, 95% CI 0.1–2.0, P = 0.243	OR 2.3, 95% CI 0.5–11.7, P = 0.324	OR 0.3, 95% CI 0.0–2.6, P = 0.212
Cardiovascular events	OR 3.6, 95% CI 1.2–11.3, P = 0.041	OR 1.9, 95% CI 0.7–5.2, P = 0.247	OR 0.8, 95% CI = 0.1–6.1, P = 0.781	OR 1.9, 95% CI 0.6–6.4, P = 0.297	OR 1.7, 95% CI 0.4–8.6, P = 0.514	OR 1.0	OR 3.0, 95% CI 0.6–15.8, P = 0.239
Cerebrovascular events	OR 1.0	OR 4.4, 95% CI 0.3–72.2, P = 0.317	OR 1.0	OR 1.0	OR 1.0	OR 1.0	OR 1.0

*ACS* autonomous cortisol secretion, *DST* dexamethasone suppression test, *DHEAS* dehydroepiandrosterone sulphate, *LNSC* late-night salivary cortisol, *UFC* urinary-free cortisol. Differences in quantitative variables are expressed in mean differences (d) between ACS and NFAI group, and for qualitative variables differences are expressed in odds ratios (OR) and 95% confident interval (in brackets).

## Discussion

Our study confirms that, when used as single tests, plasma ACTH, LNSC, UFC and DHEA-S had poor sensitivity for the diagnosis of ACS. The combination of the four tests, however, improved diagnostic accuracy for ACS reaching an AUC in the ROC curve of 0.73. On the other hand, the diagnosis accuracy of DST for the prediction of comorbidities potentially related to ACS is low, albeit other tests routinely used for the study of AIs showed even worse performances. The association of a positive DST test with hypertension and cardiovascular events seems to increase when combined with increased LNSC levels, with the addition of ACTH, DHEA-S or UFC not improving the strength of such an association.

Several studies found that patients with AIs and elevated post-DST cortisol concentrations had worse cardiometabolic profiles and increased mortality compared with patients reaching adequate cortisol suppression after this test^11–13,17^. It is currently debated which DST threshold should be used for the diagnosis of ACS. Several studies suggested that 50 nmol/L is the most sensitive threshold to identify patients with AIs and increased cardiometabolic risk^11–15^. In this line, Morelli et al.^14^ demonstrated that in patients with AI, post-DST cortisol levels increased according to the number of chronic complications. In another study^15^, using artificial neural networks, she found that the optimal cut-off of post-DST cortisol levels for detecting patients with increased cardiovascular events was 50 nmol/L (accuracy 67.3%, AUC, 0.673). Furthermore, in another study^18^ an increased risk of cardiovascular events was observed with post-DST cortisol values above 41 nmol/L (1.5 µg/dL). Our study found that, although there were some associations between DST results and cardiometabolic comorbidities, the DST had a poor diagnostic performance for the presence of these comorbidities. This finding is in agreement with earlier studies^14,15,18^, supporting that post-DST cortisol is neither accurate enough to predict the occurrence of post-surgical hypocortisolism nor the improvement of surgical complications in patients with AIs.

The poor performance of the DST and other tests of adrenal function on the prediction of comorbidities potentially related to ACS might be explained by the multifactorial origin of these prevalent cardiometabolic disorders. Hence, ACS as a single factor, is unlikely to fully predict them especially when some factors known to increase the cardiometabolic risk such as older age^19^ and subclinical co-secretion of other hormones like aldosterone^20^ are also associated with the presence of AIs. Other factors such as obesity, which can promote hyperinsulinism and thus the development of AIs, could be indirectly associated with cortisol production as well^21^. However, until better and or reliable markers of ACS become available, the DST using the serum cortisol level > 1.8 µg/dL threshold seems the most sensitive single test to identify ACS patients at risk of cardiometabolic comorbidities. Moreover, in the presence of an elevated post-DST cortisol concentration, an elevated LNSC identifies patients at even higher cardiometabolic risk.

The performance of UFC, DHEA-S, ACTH and LNSC levels for the diagnosis of ACS was poor and, for the identification of comorbidities potentially related to ACS, were even poorer than that of DST in our study. This finding supports the recommendation of most professional societies to use the DST for the evaluation of ACS in AIs^4,7,8^. At present, UFC is not recommended for the diagnosis of ACS, given that less than 20% of patients with ACS present elevated UFC levels^5,22^. The role of DHEA-S in the diagnosis of ACS is currently controversial^23–27^. In our study, DHEA-S as a single test or in combination with DST did not achieve better diagnostic performances for comorbidities potentially related to ACS than using the DST alone. Previous studies found basal ACTH levels > 2 pmol/L in up to 50% of patients with ACS and < 2 pmol/L in as many as 20% of patients with normal cortisol metabolism, also suggesting a poor diagnostic performance for ACS^28^. We found basal ACTH levels to have a weak association with the results of the DST, but no association with cardiometabolic comorbidities. LNSC—an easy, stress-free, and cost-effective alternative to late night serum cortisol—also showed limited utility for the diagnosis of ACS as suggested by previous studies^29^. Of the tests of adrenal function studied here, LNSC levels showed the greater reliability for the diagnosis of ACS as defined by the DST test, and patients with elevated LNSC and post-DST cortisol levels were those with the worst cardiometabolic profiles. Moreover, we found that the combination of basal plasma ACTH, UFC, LNSC and DHEA-S significantly increased the diagnostic accuracy for the diagnosis of ACS compared with their use as single tests, reaching an AUC of 0.73 in the ROC curve. This is in line with the recommendation of most guidelines and experts in this field of using the combination of several hormonal parameters to evaluate the presence of ACS^2,4–10^.

Our present study, however, is not free of limitations, starting by its retrospective design. Because we only included patients in whom all the diagnostic tests had been obtained, and such a decision was made on a clinical basis by their physicians, possibility exists of a selection bias towards the inclusion of a subset of more complicated patients as higher tumour size, higher DST and lower ACTH levels were found in the inclusion population compared to the excluded patients. However, we included all consecutive patients fulfilling the inclusion criteria during the study period within a single institution, thus allowing for comparable laboratory results. We did not evaluate osteoporosis, which is a recognized comorbidity related to ACS, due to inconsistent evaluation in the medical records. Therefore, the association of the results of the different evaluated tests with osteoporosis could not be evaluated. The metabolism of dexamethasone varies widely among patients^30^. Although we excluded patients with known factors associated with false positive results in the DST such as treatment with oral hormone contraceptives or other drugs known to alter dexamethasone metabolism, alcoholism, and psychiatric illness, some of these conditions might have not been registered in the medical records, and dexamethasone levels were not routinely evaluated during the DST^31^. Furthermore, other factors could also lead to false positive results in the DST^32^. Added to this is the known variability between techniques and assay kits for cortisol assays^33^ and intra-assay variability in measurements which increases in the range of low cortisol levels. Furthermore, in our institution UFC and LNSC are measured by immunochemiluminescence, which are substandard compared with the liquid chromatography/tandem mass assays recommended nowadays^34^. This limitation is supported by the results of a recent study^35^ that demonstrated that with the use of liquid chromatography/tandem mass assays, low DHEA-S levels were associated with diabetes, an association that was lost when DHEA-S was measured by immunochemiluminescence. Future studies are needed to identify more reliable and accurate markers of cortisol autonomy. In this regard, urine metabolomics^34^ and functional imaging studies such as adrenal iodomethyl-norcholesterol scintigraphy hold promise.

## Conclusion

LNSC is the one test with the highest diagnosis accuracy for ACS identification when a positive DST is used as the gold standard for ACS diagnosis. Comorbidities potentially related to ACS cannot be predicted by any single test of adrenal function possibly translating their multifactorial nature. In fact, the association of the tests evaluated here with comorbidities potentially related to ACS was poor. As a single test, DST, had the strongest association with comorbidities potentially related to ACS. Patients with elevated DST results and elevated LNSC levels had the highest cardiometabolic risk in our cohort.

## Supplementary Information

Supplementary Information.
