# Supplementary material for: Diagnostic accuracy of the different hormonal tests used for the diagnosis of autonomous cortisol secretion
Source: Sci Rep. 2021 Oct 15;11:20539. doi: 10.1038/s41598-021-00011-4 (PMC8519913; doi:10.1038/s41598-021-00011-4)
Supplement: Supplementary file 1 — Supplementary Information. [file 41598_2021_11_MOESM1_ESM.docx]

**SUPLEMENTARY MATERIAL**

**Table 1.** Differences between the patients with adrenal incidentalomas excluded or included in the study

| **PARAMETER** | **EXCLUDED (n=512)*** | **INCLUDED (n=197)** | **P value** |
| --- | --- | --- | --- |
| CLINICAL DATA:  Age, years  Female sex  ACS-comorbidities  Diabetes  Hypertension  Dyslipidaemia  Obesity  Cerebrovascular disease  Cardiovascular disease  Body mass index (kg/m^2^)  ANALYTICAL DATA:  Fasting glucose, nmol/L (mg/dl)  HbA1c (%)  LDL-c, nmol/L (mg/dl)  HDL-c, nmol/L (mg/dl)  Triglycerides, nmol/L (mg/dl)  DST, nmol/L(µg/dL)  UFC, nmol/24h (µg/24h)  ACTH, pmol/L (pg/ml)  DHEA-S (ng/ml)  LNSC, nmol/L(µg/dl)  RADIOLOGICAL DATA:  Tumour size (mm)  Bilaterality  Tumour rich in lipidic content | 63.0±11.0  55.7% (n=227)  89.6% (n=423)  25.0% (n=128)  52.0% (n=266)  50.2% (n=257)  31.6% (n=162)  1.6% (n=8)  12.8% (n=65)  29.3±5.35  5.99±1.8 (108.0±32.44)  6.2±0.9  30.7±9.3 (118.0±35.88)  13.5±3.8 (52.0±14.58)  1.28±0.7 (112.6±56.6)  49.7±49.1 (1.8±1.78)  1230.3±2107.6 (44.6±76.4)  4.7±5.9 (21.3±27.0)  651.0 [IQR=150-1750]  107.6±124.1 (3.9±4.5)  19.7±10.0  27.2% (n=139)  83.1% (n=355) | 64.5±10.1  57.4% (n=113)  85.9% (n=152)  22.3% (n=44)  57.9% (n=114)  49.0% (n=96)  31.0% (n=61)  1.0% (n=2)  10.7% (n=21)  30.3±6.3  5.87±1.6 (105.7±28.9)  6.2±0.9  30.03±8.3 (115.5±31.8)  13.96± 4.6 (53.7±17.7)  1.17±0.6 (110.2±51.1)  66.2±74.5 (2.4±2.7)  1092.41±791.1 (39.6±28.7)  3.6±2.6 (16.3±11.6)  596.2 [IQR=150-2840]  110.3±118.6 (4.0±4.3)  22.2±10.5  30.0% (n=59)  85.2% (n=132) | 0.099  0.683  0.948  0.458  0.157  0.772  0.862  0.580  0.442  0.086  0.387  0.953  0.461  0.270  0.612  0.001  0.507  0.024  0.572  0.850  0.016  0.457  0.559 |

*Patients with overt Cushing syndrome, primary aldosteronism, adrenal carcinoma, pheochromocytoma and adrenal metastasis were not included in this group

ACTH, adrenocorticotrophin; DHEA-S, dehydroepiandrosterone-sulphate; DST, dexamethasone suppression test; LNSC, late-night salivary cortisol; UFC, urinary free cortisol.
